# iProDNA-CapsNet: identifying protein-DNA binding residues using capsule neural networks

**DOI:** 10.1186/s12859-019-3295-2

**Published:** 2019-12-27

**Authors:** Binh P. Nguyen, Quang H. Nguyen, Giang-Nam Doan-Ngoc, Thanh-Hoang Nguyen-Vo, Susanto Rahardja

**Affiliations:** 1 School of Mathematics and Statistics, Victoria University of Wellington, Gate 7, Kelburn Parade, Wellington, 6140 New Zealand; 2 School of Information and Communication Technology, Hanoi University of Science and Technology, 1 Dai Co Viet, Hanoi, 100000 Vietnam; 3 School of Marine Science and Technology, Northwestern Polytechnical University, 127 West Youyi Road, Xi’an, 710072 China

**Keywords:** Protein-DNA interaction, Residue, Prediction, PSSM, Capsule neural network, Deep learning

## Abstract

**Background:**

Since protein-DNA interactions are highly essential to diverse biological events, accurately positioning the location of the DNA-binding residues is necessary. This biological issue, however, is currently a challenging task in the age of post-genomic where data on protein sequences have expanded very fast. In this study, we propose iProDNA-CapsNet – a new prediction model identifying protein-DNA binding residues using an ensemble of capsule neural networks (CapsNets) on position specific scoring matrix (PSMM) profiles. The use of CapsNets promises an innovative approach to determine the location of DNA-binding residues. In this study, the benchmark datasets introduced by Hu et al. (2017), i.e., PDNA-543 and PDNA-TEST, were used to train and evaluate the model, respectively. To fairly assess the model performance, comparative analysis between iProDNA-CapsNet and existing state-of-the-art methods was done.

**Results:**

Under the decision threshold corresponding to false positive rate (FPR) ≈ 5%, the accuracy, sensitivity, precision, and Matthews’s correlation coefficient (MCC) of our model is increased by about 2.0%, 2.0%, 14.0%, and 5.0% with respect to TargetDNA (Hu et al., 2017) and 1.0%, 75.0%, 45.0%, and 77.0% with respect to BindN+ (Wang et al., 2010), respectively. With regards to other methods not reporting their threshold settings, iProDNA-CapsNet also shows a significant improvement in performance based on most of the evaluation metrics. Even with different patterns of change among the models, iProDNA-CapsNets remains to be the best model having top performance in most of the metrics, especially MCC which is boosted from about 8.0% to 220.0%.

**Conclusions:**

According to all evaluation metrics under various decision thresholds, iProDNA-CapsNet shows better performance compared to the two current best models (BindN and TargetDNA). Our proposed approach also shows that CapsNet can potentially be used and adopted in other biological applications.

## Introduction

In biochemistry, the protein-DNA interaction is considered to be one of the vital activities that has strong impacts on diverse biological events including DNA synthesis, transcription, splicing, and restoration [1–3]. Therefore, high precision in determining the protein-DNA binding residues is crucial not only for protein function analysis but also for novel drug discovery [4]. For years, a lot of studies have been conducted to gain more understandings about the natural mechanism of protein-DNA interactions [5, 6]. Besides, to make an empirical confirmation on protein-DNA interaction, many high-throughput experimental advances have been designed such as protein microarray assays [7], ChIP-Seq [8], and protein binding microarray (PBM) [9]. Nevertheless, the identification of protein-DNA binding residues using experimental advances usually brings a great burden of cost and time. Since handling on experimental assays is complicated, using computation advances to identify DNA-binding residues is now preferable. On the other hand, the ceaselessly increasing number of unannotated protein sequences has motivated researchers to find better tools for this biological problem. Thus, employing computational models to predict the locations of the protein-DNA binding residues has become one of the most concerned topics in bioinformatics [1, 5, 10]. In the last decade, a number of computational models have been developed to identify DNA-binding residues [1, 11]. Based on the used features, these methods can be classified into three major groups: (i) structure-based models [12, 13], (ii) sequence-based models [10, 14], and (iii) hybrid models (using both sequence and structural features) [15].

In general, the structure-based and hybrid models frequently come up with better prediction accuracies compared to sequence-based models due to efficient exposure of specific distinctions between DNA-binding and non-binding residues [15]. The B-factor, surface curvature, and depth index (DPX), for instances, are three of numerous structure-based features that have been vastly employed to identify DNA-binding residues [15] with fairly good performances. Structure-based and hybrid models, however, also find more difficulties in circumstances in which no defined 3D-structure proteins are available. This situation is common for newly investigated proteins with only peptide sequences being determined because performing 3D-structural reconstruction for a particular protein usually time-consuming. Despite being supported by some popular homology modeling tools (e.g., MODELLER [16] and I-TASSER [17]), 3D-structural reconstruction for a new protein is still not a simple work because of large structural inconsistencies between the computer-aided rebuilt structure and the actual one, especially when appropriate structural templates are unavailable [18]. Moreover, the fast ever-growing genome sequencing technologies also add more distance between a number of protein sequences and their rebuilt structures. Hence, using sequence-based computational models to identify DNA-binding residues seems to be more realistic and reasonable to meet the needs.

In comparison with structure-based methods, sequence-based models do not require protein structural information to predict DNA-binding residues. The last decade has seen significant growth in machine learning-based models (e.g., DNABR [19], DP-Bind [12], BindN [10], and MetaDBsite [6]) used for prediction of DNA-binding residues based on given sequences. These sequence-based methods use only protein sequence information to identify DNA-binding residues under the support of several common learning algorithms including Random Forest (RF) [20], Support Vector Machine (SVM) [21], k-Nearest Neighbors (k-NN) [22], and Extreme Gradient Boosting (XGBoost) [23]. In 2006, Wang et al. proposed BindN [10], a prediction model using the SVM algorithm receiving sequence features comprising of the hydrophobicity index, the molecular mass of a residue, and the pKa value of the side chain as model inputs. A year later, DP-Bind [14] developed by Hwang et al. was introduced as a web-based prediction tool that combined three learning algorithms encompassing kernel logistic regression, penalized logistic regression and SVM to improve the performance. This tool ultilizes the position-specific scoring matrix (PSSM) profiles generated from protein sequences. In 2015, Wong et al. published a new computational model that utilized not only protein sequences but also DNA sequences to enhance feature specificity to predict possible interactions between a nucleotide and protein residues from distinctive defined DNA-binding domain families [24]. Additionally, Wong et al. also proposed the kmerHMM [25] - a hidden Markov model (HMM) utilizing belief propagations. This model is capable of adapting and converting protein binding microarray raw data into another form so-called median-binding intensities of single k-mers to recognize DNA motifs. Although these existing models have come up with certain achievements, further studies for model improvement is still needed.

Using machine learning algorithms to construct prediction models for protein-DNA binding residues is not straightforward due to the inherent data imbalance in the residues. In fact, the number of non-binding residues is predominant over that of DNA-binding residues. Therefore, using resampling techniques is currently the most frequent solution for class imbalance [26, 27]. In this scenario, over-sampling and under-sampling are the two most commonly applied techniques as described in previous studies [26–28]. Over-sampling expands the training dataset and hence training time and predicting time usually elongate. Additionally, this technique is often claimed to cause the over-fitting problem. On the contrary, under-sampling reduces the training dataset and therefore leads to implicit risk of feature losses or weak feature characterization. In 2016, Hu et al. suggested a prediction model with a solution for the class imbalance. They proposed combining an under-sampling technique and a suitable boosting ensemble algorithm. Then, their proposed ensembled learning model was constructed using various distinctive classifiers on the modified balanced dataset [29].

In this study, we applied the capsule neural network (CapsNet) architecture [30], one of the latest deep learning approaches, on the PSSM features generated from the training and test datasets introduced by Hu et al. [29]. PSSM has been shown to be a suitable data representation for applying deep learning architectures, especially convolutional neural networks (CNNs), on various bioinformatics problems in general and on binding prediction problems in particular [31]. CapsNet is an advanced deep learning architecture; however, it has not been widely applied in bioinformatics except for a recent work on prediction of protein post-translational site modification [32]. Compared to CNN architectures having similar computation costs, CapsNet often archives better performance [30]. On small training datasets, CapsNet also outperforms CNNs as the result of having ability to characterize hierarchical relationships between simple and complex features [30, 32]. We anticipated using CapsNet with PSSM features would outperform other algorithms which have been successfully used in prediction of protein-DNA binding residues. Our method used 10-fold cross-validation and trained 10 CapsNet models from 10 sub-training datasets. To deal with the class imbalance issue, random under-sampling (RUS) [33] was applied on each sub-training dataset. Eventually, these 10 CapsNet models were ensembled and fed with the test dataset to obtain the final testing results. For a fair assessment, we compared our proposed approach with other state-of-the-art methods using the same test dataset.

## Materials and methods

### Benchmark datasets

For model construction and evaluation, we used PDNA-543 and PDNA-TEST as the training dataset and the independent test dataset, respectively. These two datasets resemble those used in the TargetDNA method [29]. Totally there are 584 non-redundant protein sequences obtained after removing redundant sequences using the CD-hit software [34] with the identity threshold of 30%. The training dataset has 543 protein sequences while the independent test dataset has 41 protein sequences. The training dataset consists of DNA-binding residues (positive samples) and 134,995 non-binding residues (negative samples). In the test dataset, there are 734 positive samples and 14,021 negative samples. The detailed information of PDNA-543 and PDNA-TEST is summarized in Table 1.

**Table 1:** Data distribution in the training set (PDNA-543) and the independent testing set (PDNA-TEST)

Dataset	No. of Sequences	No. of Positive Samples (*a*)	No. of Negative Samples (*b*)	Ratio (*a*/*b*)
PDNA-543	543	9,549	134,995	14.137
PDNA-TEST	41	734	14,021	19.102

### Feature representation

The position-specific scoring matrix (PSSM) has been widely used to extract features from protein sequences. We used the PSI-BLAST (Position-Specific Iterative Basic Local Alignment Search Tool) [35] against the Swiss-Prot database [36] with three iterations and a cut-off E-value of 0.01 to generate a PSSM profile from an input protein sequence. In the generated matrix, each of *L* rows represents the corresponding amino acid in the input protein sequence with length *L*, and each of 20 columns represents a particular amino acid among the total of 20 standard amino acids building up the protein structure. Equation (1) shows how the PSSM profile with respect to a protein *P* with *L* amino acids being calculated.

1 $$  P_{PSSM} = \left[\begin{array}{cccccc} E_{1\rightarrow1} & E_{1\rightarrow2} & \cdots & E_{1\rightarrow j} & \cdots & E_{1\rightarrow20}\\ E_{2\rightarrow1} & E_{2\rightarrow2} & \cdots & E_{2\rightarrow j} & \cdots & E_{2\rightarrow20} \\ \vdots & \vdots & \cdots & \vdots & \cdots & \vdots \\ E_{i\rightarrow1} & E_{i\rightarrow2} & \cdots & E_{i\rightarrow j} & \cdots & E_{i\rightarrow20} \\ \vdots & \vdots & \cdots & \vdots & \cdots & \vdots \\ E_{L\rightarrow1} & E_{L\rightarrow2} & \cdots & E_{L\rightarrow j} & \cdots & E_{L\rightarrow20} \\ \end{array}\right],  $$

where *E*_*i*→*j*_ is the score of the mutation from an amino acid in the *i*^*th*^ position of the protein sequence to the standard amino acid *j* ($j = \overline {1,20}$) during evolution. Positive scores suggest the mutation *E*_*i*→*j*_ happens more often than expected by chance while negative scores indicate the opposite. Then each score *x* in the PSSM profile is rescaled to the interval (0,1) using the standard logistic function:

2 $$  f(x) = \frac{1}{1+e^{-x}}.  $$

Then, the window sliding technique was applied to the rescaled PSSM to obtain the PSSM feature vector for each amino acid since the PSSM score of a particular amino acid and its neighbors may affect the DNA-binding ability. We set the window size to 21, leading to the size of 21×20 for each PSSM feature vector.

### Model architecture

**Fig. 1:**
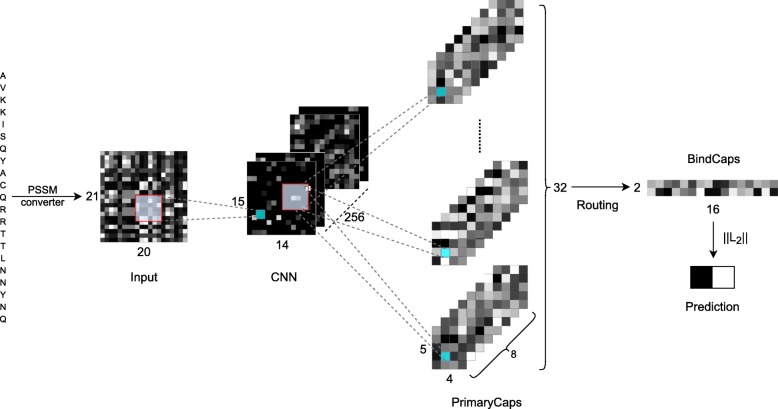
Architecture of the proposed CapsNet model

The architecture of our proposed CapsNet model, as shown in Fig. 1, consists of two 2-dimensional convolutional layers (CNN and PrimaryCaps) and one fully connected layer (BindCaps). The first layer, CNN, detects basic features of the input PSSM corresponding to a protein sequence. The 21×20 PSSM is convolved with 256 filters of size 7×7 at stride 1 with ReLU activation function to produce a 15×14×256 tensor. For improving the training speed, performance, and stability of the CapsNet model and preventing overfitting, we added a batch normalization sub-layer [37] and a dropout sub-layer [38] with the neuron dropping rate of 0.7 at the end of the first layer.

The second layer, PrimaryCaps, consists of 32 primary capsules which combines the basic features detected in the first layer. This is done by the use of 8 filters with the size of 7×7×256 with stride 2 in each capsule that takes the 15×14×256 tensor from the CNN layer as input and produces a 5×4×8 output tensor. Here, 8 is the dimension of the capsule vectors in PrimaryCaps which is similar to that in the original CapsNet architecture [30]. Since there are 32 capsules, the shape of the output of this layer is 5×4×8×32. A batch normalization sub-layer is included with a dropout sub-layer with the neuron dropping rate of 0.2 at the end of PrimaryCaps. A non-linear “squashing” function is used to scale the length of the output vector of each capsule to [0, 1] since it is the probability that the current input represents the encoded entity:

3 $$ \mathrm{v}_{j} = \frac{{\left\| {\mathrm{s}_{j}} \right\|^{2} }}{{1 + \left\| {\mathrm{s}_{j}} \right\|^{2} }}\frac{{\mathrm{s}_{j} }}{{\left\| {\mathrm{s}_{j}} \right\|}},  $$

where v_*j*_ is the vector output of capsule *j* and s_*j*_ is its input.

**Fig. 2:**
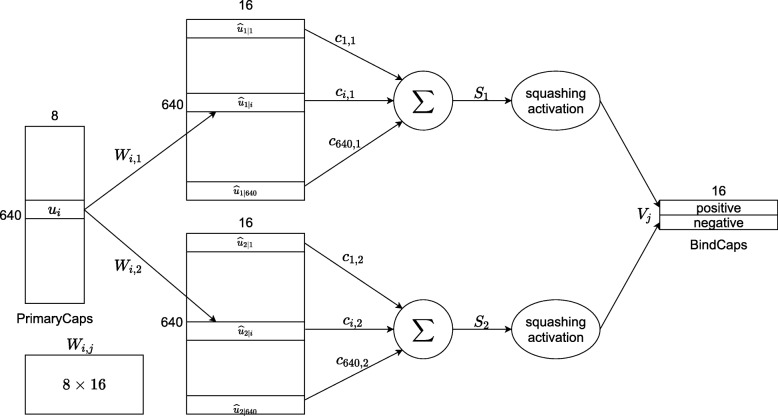
Computation between two layers: PrimaryCaps and BindCaps

The next layer, BindCaps, has two 16-dimensional “binding” capsules corresponding to 2 possible labels of the input protein sequence: positive and negative (indicating whether protein-DNA binding exists at the centered amino acid or not). The input of each capsule in this layer is a 5×4×8×32 tensor. In other words, they are 5×4×32 8-dimensional vectors, each is assigned with an 8×16 weight matrix which then multiplies with an 8-dimensional input vector to produce a 16-dimensional vector. These 16-dimensional vectors are weighted (the weights are determined by the dynamic routing algorithm) and summed over, and the results are then passed through the squashing function to produce two 16-dimensional vectors as the output. The computation between the PrimaryCaps and BindCaps layers is illustrated in Fig. 2 and the complete dynamic routing algorithm is described in [30]. There are 640 8-dimensional capsules (each *u*_*i*_ is an 8-D vector) in PrimaryCaps. Each is produced by multiplying *u*_*i*_ by a weight matrix *W*_*i*,*j*_ (size of 8×16). Capsule vector *V*_*j*_ (*j* = 1 or 2) in BindCaps is a 16-dimensional vector which is computed by passing the weighted sum over all output $\hat u_{j\left | i \right.} $ from PrimaryCaps through the squashing function. Parameter *c*_*i*,*j*_ is determined by the iterative dynamic routing process. Similar to the previous two layers, a batch normalization sub-layer and a dropout sub-layer with the neuron dropping rate of 0.1 are included at the end of BindCaps. The L2-norms of the two 16-dimensional vectors are then computed to obtain the final output of the CapsNet model as a 2-dimensional vector.

The loss function for training our CapsNet model is the sum of two separate margin losses, *L*_*k*_ for each “binding” capsule, *k*:

4 $$ {} \begin{aligned} L_{k} &= T_{k} \max (0,0.9 - \left\| {\mathrm{v}_{k}} \right\|)^{2}\\ &\quad+ 0.5(1 - T_{k})\max (0,\left\| {\mathrm{v}_{k}} \right\| - 0.1)^{2}, \end{aligned}  $$

where v_*k*_ is the output of “binding” capsule *k* and *T*_*k*_=1 if protein-DNA binding exists.

The architecture of our CapsNet model is relatively similar to the encoder in the original capsule neural network [30]. In addition to the differences in the number filters in each layer, we decreased the filter size in the first two layers from 9×9 to 7×7 and included batch normalization and dropout in all the layers. Our preliminary experimental results confirmed that reducing filter size and including dropout helped the model to be less prone to overfitting and integrating batch normalization improved validation performance.

### Model training and testing

**Fig. 3:**
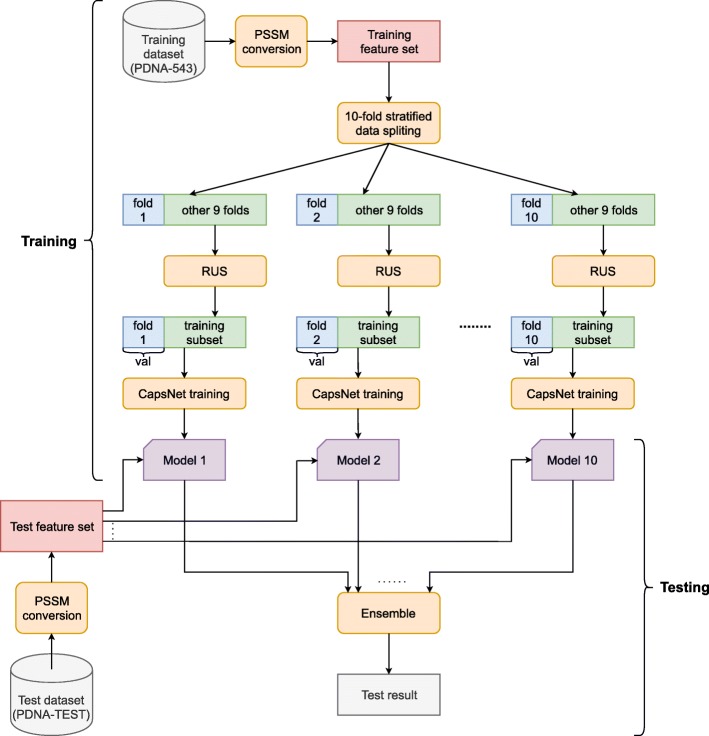
Diagram of training and testing the CapsNet models

The diagram of training and testing our model is described in Fig. 3. First, PSSM features are extracted from both the training set (PDNA-543) and the test set (PDNA-TEST) to form the training feature set and the test feature set, respectively. The training feature set is then divided into 10 mutually exclusive stratified folds, and they are combined to form 10 combinations of 9 training folds and 1 validation fold. For each of the combinations, a CapsNet model was trained using a balanced training subset created from the 9 training folds using random under-sampling (RUS) [33], and the model was optimized and validated on the validation fold. Adam optimization algorithm [39] was used along with each minibatch of 256 samples. Under the learning rate of 0.0001, the model was trained with a maximum of 300 epochs. During the training iteration, the early stopping strategy was used in such the way that if no improvement in validation loss after 20 consecutive epochs, the training process would be automatically terminated. The learning rate would be halved whenever the validation loss did not improve after 10 consecutive epochs. The number of iterations used in the routing algorithm was set to 3 (by default) and the margin loss function was employed. The best model was saved at the end of the training process. During testing, these 10 trained CapsNet models were fed with the test feature set and then the final testing results were calculated by averaging the predictions from all the models and compared with the truth labels.

In our experiments, all the deep-learning models were implemented using Keras 2.2.4 and TensorFlow 1.13.1. Model training and testing were performed on an i5 9600k workstation with the Ubuntu 18.04.1 LTS operating system and equipped with 16GB RAM and one GPU NVIDIA GTX 1080Ti. It took about 6 seconds to train 1 epoch and 43 seconds to complete testing.

### Cross-Validation

**Fig. 4:**
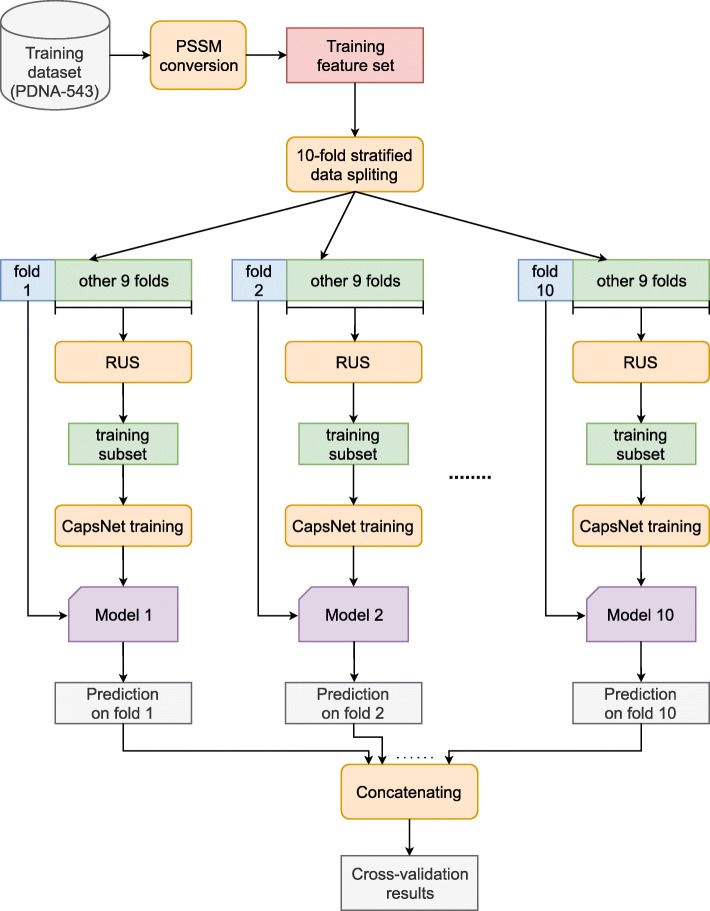
10-fold cross-validation process

In order to compare our framework with other related methods, we also performed 10-fold cross-validation on the training dataset (PDNA-543). As shown in Fig. 4, the model training process in this case is somewhat different from that in “Model training and testing” section. First, we extracted the PSSM feature set from the PDNA-543 dataset. Then, the feature set was randomly split into 10 mutually exclusive folds using stratified sampling. Each fold was in turn used as the validation set while the remaining 9 folds were use as the training set for training a CapsNet model. RUS was then applied to the training set for rebalancing whereas the validation set was left intact. The model was trained using the balanced 9-fold training dataset for 100 epochs and then tested once on the validation fold to obtain the predictions on that fold. This process was repeated for 10 times with all 10 different validation folds to produce 10 different prediction arrays. These prediction arrays were concatenated for the predictions on the whole PDNA-543 dataset and used to compare with the truth labels to produce the cross-validation results.

### Evaluation metrics

In this study, five evaluation metrics, including Sensitivity (SN), Specificity (SP), Accuracy (ACC), Precision (PR), and Matthews’s correlation coefficient (MCC) were used to evaluate the model performance. These mathematical expressions of these evaluation metrics are specified below:

5 $$ {}Accuracy \,(ACC) = \frac{{TP+TN}}{{TP + TN + FP + FN}}  $$

6 $$ {}Sensitivity \,(SN) = \frac{{TP}}{{TP + FN}}  $$

7 $$ {}Specificity \,(SP) = \frac{{TN}}{{TN + FP}}  $$

8 $$ {}Precision \,(PR) = \frac{{TP}}{{TP + FP}}  $$

9 $$ {}MCC \,=\, \begin{aligned} \frac{{TP \times TN - FP \times FN}}{{\sqrt {(TP + FP)(TP + FN)(TN + FP)(TN + FN)} }} \end{aligned}  $$

where TP, FP, TN, and FN are abbreviated terms of True Positive, False Positive, True Negative, and False Negative values, respectively. These evaluation metrics, however, changes under the adjustment of the decision threshold when making prediction. To fairly compare our proposed approach with state-of-the-art methods, we set the threshold in the same way as in [29], i.e., select the threshold so that we have the following cases: (i) FPR (False Positive Rate, which is equal to 1 - Specificity) ≈ 15%, (ii) FPR ≈ 5%, and (iii) SN ≈ SP (Sensitivity is approximately equal to Specificity) during cross-validation and testing. We also set the threshold so that FPR ≈ 8% when testing. In addition, since the Area Under the Curve (AUC) of the Receiver Operating Characteristic (ROC) curve is independent on the threshold, we also used AUC as an important metric to evaluate the performance of our models. Higher AUC values mean better performances.

## Results and discussion

### Cross-Validation and model evaluation

**Fig. 5:**
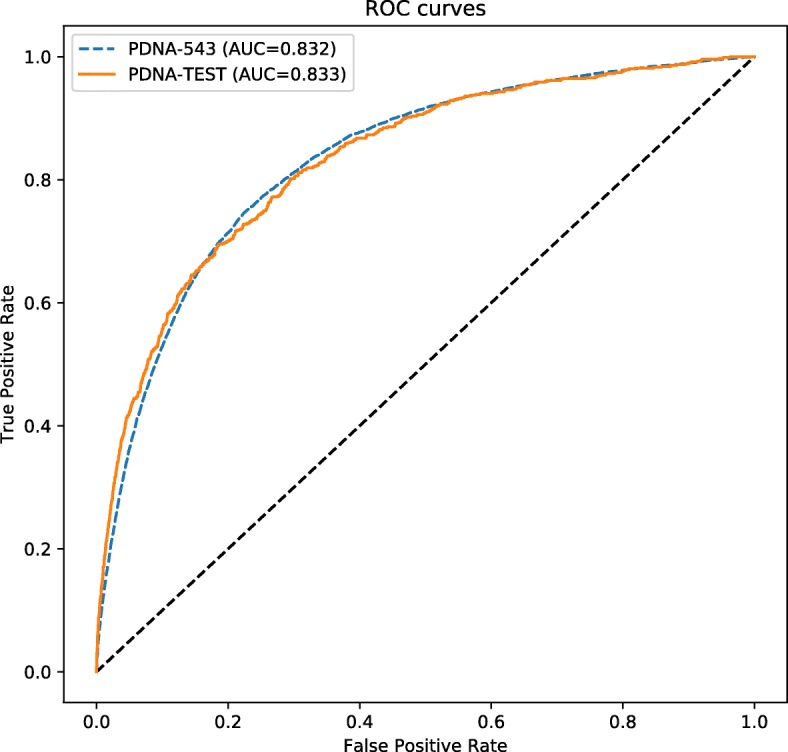
ROC curves for iProDNA-CapsNet on PDNA-543 (blue dashed line) in model testing and on PDNA-TEST (orange solid line) in 10-fold cross-validation

Tables 2 and 3 show the performances of our models on the training dataset (PDNA-543) using 10-fold cross-validation and on the test dataset (PDNA-TEST), respectively, under four different settings of the decision threshold including (i) default threshold = 0.5, (ii) FPR ≈ 5% (SP ≈ 0.95), (iii) FPR ≈ 15% (SP ≈ 0.85), and (iv) SN ≈ SP. The agreement between cross-validation outcomes and testing outcomes under the four different settings shows that our models are good at generalization. This is also confirmed by the two almost-identical ROC curves in Fig. 5.

**Table 2:** 10-fold cross-validation performances of iProDNA-CapsNet on the training dataset (PDNA-543) under various decision thresholds

Setting	ACC (%)	SN (%)	SP (%)	PR (%)	MCC	AUC
Threshold = 0.5	74.73	**77.38**	74.55	17.32	0.282	0.832
FPR ≈ 5%	**91.21**	36.31	**95.00**	**33.34**	0.301	0.832
FPR ≈ 15%	83.66	64.21	85.00	22.78	**0.313**	0.832
SP ≈ SN	76.02	76.02	76.02	17.93	0.287	0.832

Values which are significantly higher than the others are in bold

**Table 3:** Performances of iProDNA-CapsNet on the test dataset (PDNA-TEST) under various decision thresholds

Setting	ACC (%)	SN (%)	SP (%)	PR (%)	MCC	AUC
Threshold = 0.5	75.72	74.79	75.77	13.59	0.245	0.833
FPR ≈ 5%	**92.38**	42.17	**94.93**	**29.78**	**0.315**	0.833
FPR ≈ 8%	91.13	45.73	93.45	26.23	0.302	0.833
FPR ≈ 15%	84.05	65.38	85.00	18.17	0.285	0.833
SP ≈ SN	75.34	**75.36**	75.34	13.47	0.245	0.833

Values which are significantly higher than the others are in bold

For the PDNA-543 dataset, among the four settings, the accuracy when FPR ≈ 5% holds the first place, followed by those when FPR ≈ 15%, SP ≈ SN, and the default threshold. The specificity and precision when FPR ≈ 5% are also significantly higher than those of other thresholds. When FPR ≈ 5%, the accuracy, specificity, and precision are increased by about 9.0–22.0%, 12.0–27.0%, and 46.0–92.0%, respectively, compared to the other setups. The MCC of the model increases following the decrease in FPR and varies between 0.282 and 0.313. Under the threshold corresponding to FPR ≈ 15%, the MCC is higher than the other setups. In contrast, using the default threshold leads to the highest value of sensitivity.

For the PDNA-TEST dataset, under the threshold corresponding to FPR ≈ 5%, the accuracy, specificity, precision, and MCC come up with significantly higher values compared to other setups. The accuracy drops by about 10.0%, 22.0%, and 22.0% when changing the setting from FPR ≈ 5% to FPR ≈ 15%, SP ≈ SN, and the default threshold, respectively. Using the threshold corresponding to SP ≈ SN returns higher sensitivity compared to other setups but not significantly different from using the default threshold due to only small adjustment between sensitivity and specificity.

Besides, we also set another threshold so that FPR ≈ 8% in order to observe possibly new trend of change. This setting gives similar performance compared to the case when FPR ≈ 5% with smaller values of all the metrics except sensitivity.

### Comparative analysis

Table 4 shows the performance of our models compared with that of other state-of-the-art methods (data excerpted from [29]) including BindN [10], ProteDNA [40], MetaDBSite [6], DP-Bind [14], DNABind [41], BindN+ [42], and TargetDNA [29]. All the methods were tested on the same test dataset (PDNA-TEST), and among those methods, only BindN+ and TargetDNA provided performance information with two settings corresponding to FPR ≈ 5% and FPR ≈ 15%.

**Table 4:** Performance comparison between iProDNA-CapsNet and other state-of-the-art methods

Method	Setting	ACC (%)	SN (%)	SP (%)	PR (%)	MCC
BindN	Unknown	79.15	45.64	80.90	11.12	0.143
ProteDNA	Unknown	95.11	4.77	99.84	60.30	0.160
MetaDBSite	Unknown	90.41	34.20	93.35	21.22	0.221
DP-Bind	Unknown	81.40	61.72	82.43	15.53	0.241
DNABind	Unknown	79.78	70.16	80.28	15.70	0.264
BindN+	FPR ≈ 5%	91.58	24.11	**95.11** ^*a*^	20.51	0.178
	FPR ≈ 15%	83.69	50.81	85.41	15.42	0.213
TargetDNA	FPR ≈ 5%	90.89	**45.50** ^*a*^	93.27	26.13	0.300
	FPR ≈ 15%	**84.52** ^*b*^	60.22	**85.79** ^*b*^	18.16	0.269
iProDNA-CapsNet	FPR ≈ 5%	**92.38** ^*a*^	42.17	94.93	**29.78** ^*a*^	**0.315** ^*a*^
	FPR ≈ 15%	84.05	**65.38** ^*b*^	85.00	**18.17** ^*b*^	**0.285** ^*b*^

Values which are significantly higher than the others are in bold with ^*a*^FPR ≈ 5% and ^*b*^FPR ≈ 15%

Under the threshold corresponding to FPR ≈ 5%, the accuracy, sensitivity, precision, and MCC of our model increases by about 2.0%, 2.0%, 14.0%, and 5.0% with respect to TargetDNA and 1.0%, 75.0%, 45.0%, and 77.0% with respect to BindN+, respectively. In comparison with BindN+, our model come up with a significant improvement in precision (45.0%) and MCC (about 77.0%) and these surges are very meaningful to indicate the small variation among different-run values as well as high stability of our model. In comparison with TargetDNA, our model’s precision remarkably rises by 14.0% and this outgrowth reflects the considerable decline in variation among the different-run values. Besides, the specificity among methods in comparison is not significantly different. Therefore, under the threshold corresponding to FPR ≈ 5%, our proposed method seems to cover the weaknesses of both TargetDNA and BindN+. On the other hand, under the threshold corresponding to FPR ≈ 15%, the accuracy in our model, TargetDNA, and BindN+ all decrease by about 10.0%, 7.5%, and 9.5% and these declines are not far different from each other. Additionally, the specificity of the three models also drops in the range from 9.0% to 12.0%. In terms of precision, the fall in this metric decreases from our model (64.0%), followed by BindN+ (33.0%) and TargetDNA (27.0%). With regards to MCC, our method and TargetDNA share a common pattern of downward change, while upward change is observed in BindN+. When changing the threshold corresponding to FPR ≈ 5% to that corresponding to FPR ≈ 15%, the sensitivity of our model, TargetDNA, and BindN+ all demonstrates with a remarkable growth by about 65.0%, 80.0%, and 100.0%, respectively, as a result of a trade-off between sensitivity and specificity.

Among the methods with unknown threshold settings, ProteDNA [40] is the only one having higher accuracy and precision compared to our model. However, our model’s MCC is twice as high as ProteDNA’s MCC. Given the crucial role of MCC over other metrics as confirmed [43], we can therefore take that our model remains its competitive role with high stability. For the rest of the other approaches including MetaDBSite [6], DP-Bind [14], and DNABind [41]), our proposed method also shows a significant improvement in accuracy, precision, and MCC. The sensitivity of our model under the threshold corresponding to FPR ≈ 5% is about 900.0% and 125.0% higher than ProteDNA and MetaDBSite respectively while this metric under the threshold corresponding to FPR ≈ 15% notably grown by about 150.0%, >1,300.0%, 200.0%, and 6.0% compared to BindN, ProteDNA, MetaDBSite, and DP-Bind. The precisions of our model under the threshold corresponding to FPR ≈ 5% and the threshold corresponding to FPR ≈ 15% are remarkably higher than those of other methods. The improvement in precision fluctuated from roughly 140.0% (compared to MetaDBSite) to 270.0% (compared to BindN). Although ProteDNA has been reported to have an accuracy of 95.11%, a specificity of 99.84%, and a precision of 60.30%. Among the three reported metrics of ProteDNA, only its precision is far higher than that of our model but its very low MCC has weakened the trust-ability of this model. Among all the methods, our model obtained a meaningfully higher MCC which are boosted from about 8.0% (compared to DNABind) to 220.0% (compared to ProteDNA).

Our method, iProDNA-CapsNet, achieves very good values for MCC [43], which is an important metric whose crucial role has been far confirmed. Among the common evaluation metrics, MCC is the only one that uses up all information of the confusion matrix. Moreover, with respect to imbalanced datasets, MCC is the most important and informative metric that correctly assesses whether a prediction model is stable and robust while a single accuracy metric would not be sufficient to determine that status. With this dataset, MCC is therefore the most important metric. Eventually, under the threshold corresponding to FPR ≈ 5% and FPR ≈ 15%, our model shows its superior performance and high stability compared to other methods.

### Software availability

We deployed our model to an user-friendly and freely accessible web server at https://github.com/ngphubinh/iProDNA-CapsNet. Users can easily submit a protein sequence in FASTA format and receive the prediction result of protein-DNA binding residues in the sequence. We provided four different settings of the decision threshold as specified in this manuscript, including the (i) default threshold = 0.5, (ii) FPR ≈ 5% (SP ≈ 0.95), (iii) FPR ≈ 15% (SP ≈ 0.85), and (iv) SN ≈ SP. The procedure to predict protein-DNA binding residues starts on our web server when there is a query protein sequence submitted in the FASTA format along with a decision threshold and an optional email address. The corresponding PSSM profile is then extracted by PSI-BLAST incorporated into our server, and subsequently the PSSM feature set is derived by placing a sliding window on each amino acid in the sequence (with zero padding in some of the first and last amino acids). Finally, the feature set is submitted to our iProDNA-Capsnet model for prediction, and the results will be sent back to users. When viewing the result page, users can choose a desired decision threshold from a list ranging from 0.05 to 0.95 with the step of 0.05 to refine the prediction result on the submitted protein sequence.

## Conclusions

In this paper, a novel deep learning framework - CapsNet combining with PSSM features is proposed for the prediction of protein-DNA binding residues. iProDNA-CapsNet has significantly better performance than the state-of-the-art methods. In particular, the robustness and efficiency of iProDNA-CapsNet have been demonstrated by the remarkable improvement in most of the evaluation metrics, especially for MCC and accuracy. Additionally, the application of a new deep learning architecture so-called CapsNet to address this biological issue opens a new direction in similar topics, for example, RNA-protein binding [44] and ATP-protein bindings [45].

## Data Availability

The benchmark dataset used in this study were collected from the previous work by Hu et al., 2017. The benchmark dataset were downloaded and processed under the instruction described in the paper entitled “Predicting Protein-DNA Binding Residues by Weightedly Combining Sequence-Based Features and Boosting Multiple SVMs” by Hu et al. (10.1109/TCBB.2016.2616469). A web server implementing the proposed method is available at https://github.com/ngphubinh/iProDNA-CapsNet.

## Abbreviations

ATP: Adenosine triphosphate
AUC: Area under the ROC curve
CapsNets: Capsule neural networks
CNN: Convolutional neural network
DNA: Deoxyribonucleic acid
FPR: False positive rate
HMM: Hidden Markov model
k-NN: k-nearest neighbors
MCC: Matthew’s correlation coefficient
PSI-BLAST: Position-specific iterative basic local alignment search tool
PSMM: Position specific scoring matrix
ReLU: Rectified linear unit
RF: Random forest
RNA: Ribonucleic acid
ROC: Reciever operating characteristic
RUS: Random under-sampling
SVM: Support vector machine
XGBoost: Extreme gradient boosting
